# Effects of Plant Extracts on Dentin Bonding Strength: A Systematic Review and Meta-Analysis

**DOI:** 10.3389/fbioe.2022.836042

**Published:** 2022-02-24

**Authors:** Shikai Zhao, Fang Hua, Jiarong Yan, Hongye Yang, Cui Huang

**Affiliations:** ^1^ The State Key Laboratory Breeding Base of Basic Science of Stomatology (Hubei-MOST) and Key Laboratory for Oral Biomedical Ministry of Education, School and Hospital of Stomatology, Wuhan University, Wuhan, China; ^2^ Department of Orthodontics, Center for Evidence-Based Stomatology, School and Hospital of Stomatology, Wuhan University, Wuhan, China; ^3^ Division of Dentistry, School of Medical Sciences, Faculty of Biology, Medicine and Health, Manchester Academic Health Science Centre, University of Manchester, Manchester, United Kingdom; ^4^ Department of Prosthodontics, School and Hospital of Stomatology, Wuhan University, Wuhan, China

**Keywords:** dentin, bonding, plant extracts, natural crosslinkers, adhesives, primers

## Abstract

**Objective:** To systematically review *in vitro* studies that evaluated the effects of plant extracts on dentin bonding strength.

**Materials and Methods:** Six electronic databases (PubMed, Embase, VIP, CNKI, Wanfang and The Cochrane Library) were searched from inception to September 2021 in accordance with the Preferred Reporting Items for Systematic Reviews (PRISMA). *In vitro* studies that compared the performance of dental adhesives with and without the plant extracts participation were included. The reference lists of the included studies were manually searched. Two researchers carried out study screening, data extraction and risk of bias assessment, independently and in duplicate. Meta-analysis was conducted using Review Manager 5.3.

**Results:** A total of 62 studies were selected for full-text analysis. 25 articles used the plant extracts as primers, while five added the plant extracts into adhesives. The meta-analysis included 14 articles of *in vitro* studies investigating the effects of different plant extract primers on dentin bonding strength of etch-and-rinse and self-etch adhesives, respectively. The global analysis showed statistically significant difference between dental adhesives with and without plant extract primers. It showed that the immediate bond strength of dental adhesives was improved with the application of plant extract primers.

**Conclusion:** The application of proanthocyanidin (PA) primers have positive effect on the *in vitro* immediate bonding strength of dental adhesives irrespective of etch-and-rinse or self-etch modes.

## Introduction

Dentin bonding is the foundation of esthetic restoration (Drummond, 2008). Nowadays, manufacturers claim that dental adhesive system has already developed to the eighth generation (Taneja et al., 2017). However, irrespective of acceptable immediate bonds, the long-term bonding strength of these adhesives is inadequate (Deligeorgi et al., 2001; Hass et al., 2016a). As a result, nearly half of esthetic restorations cannot serve for more than 10 years, and dentists have to spend 60% of working hours to replace them (Mjor et al., 2000; Deligeorgi et al., 2001). Thus, the improvement of long-term bond strength is still a puzzle that needs to be solved.

Unsatisfactory long-term dentin bonds are usually attributed to two reasons: The degradation of dentin collagen within the hybrid layer; and the emergence of secondary caries at the interface (Brackett et al., 2011). A reasonable strategy to solve these problems is to modify contemporary dental adhesives with different additives, such as chlorhexidine, nano-silver, carbon nanotube and amorphous calcium phosphate (Carrilho et al., 2007; Borges et al., 2013; Zhang et al., 2013; Alkatheeri et al., 2015). Amongst these additives, plant extracts attracted great attention due to their biological safety and functional versatility (Gotti et al., 2015; Yang et al., 2017; Yu et al., 2017). Many articles have reported the advantages of natural plant extracts, including their capability to stabilize dentin collagen (La et al., 2009), and to inhibit MMPs (Du et al., 2012; Yang et al., 2016) and microbes (Kaul et al., 1985; Rigano et al., 2007). Therefore, many researchers have been attempting to dope plant extracts into adhesives or provide a separate plant extract primer to achieve high antibiotic property and improved long-term bond strength (La et al., 2009; Borges et al., 2013; Gotti et al., 2015; Yang et al., 2017).

However, the combination of different adhesives with different plant extracts may produce unpredictable results, and different concentration of plant extract primer may have different bonding performance (Macedo et al., 2009; Islam et al., 2014). Previous studies have tested a limited amount of plant extracts, using different experimental designs, with contradictory conclusions. Thus, a comprehensive overview summarizing the effect of all existing plant extracts on dental adhesives will be helpful for dental clinicians and relevant researchers.

The objectives of this study are to systematically review the *in vitro* studies that evaluated adhesive-dentin bond strength with or without plant extracts participation and to compare different plant extracts in terms of bond strength. The hypotheses are: no difference exists in the bond strengths when modifying the adhesives with plant extracts; no difference exists in the bond strengths when plant extract primers are used; no difference exists in the bond strength when using different concentrations of plant extracts.

## Materials and Methods

### Criteria for Considering Studies for This Review

#### Inclusion Criteria

• Studies that added plant extracts to dental adhesives or used plant extract as primers.
• Studies that compared the performance of dental adhesives with and without the participation of plant extracts.
• *In vitro* studies that evaluated the bond strength of dental adhesives.

#### Exclusion criteria

Reviews, clinical trials or case reports.

#### Search Strategy

A systematic electronic search was conducted by two independent reviewers (SZ and HY) using nine databases (PubMed, Embase, Web of Science, Cochrane Library, VIP, CNKI, Wanfang, OpenGrey literature and ProQuest Dissertation Abstracts) from inception to September 2021 to identify articles related to plant extracts and dental bonding. The search terms were a combination of subject terms and free-text terms (Appendix Table A1).

When multiple publications about the same intervention were identified, the most informative and relevant article was selected for inclusion.

### Data Collection and Analysis

#### Selection of Studies

Article titles and abstracts were independently screened by two authors (SZ and HY). The authors conducted a second review when the inclusion criteria were met. The abstracts were examined by two review authors (SZ and HY) independently using the same inclusion criteria. If there were disagreements, the abstract would be assessed by the third author (FH). Then full text of all potentially relevant studies were retrieved and independently assessed in duplicate by two review authors (SZ and HY). Any disagreement regarding the eligibility of the included studies was resolved through discussion with the third reviewer.

### Data Extraction and Management

Data extraction was performed independently by two authors (SZ and HY). The demographic data, plant extracts used, plant extract concentration, bonding systems, as well as outcomes were recorded (Table 1). If any information was missing, we contacted the corresponding authors *via* email.

**TABLE 1:** Characteristics of the included studies.

First author	Year	Country	Publication	Plant extracts	Action modes	Plant extracts concentration	Dental adhesives	Outome
Albuquerque N	2019	Brazil	Oper Dent	EGCG	Adhesive	0.1% w/v	Single Bond 2 (3M ESPE, St. Paul, MN, United States)	MTBS
Yang H	2017	China	SCI REP	Quercetin	Adhesive	100, 500 and 1,000 μg/ml	Single Bond 2	MTBS
Yu HH	2017	China	Materials (Basel)	EGCG, EGCG-3Me	Adhesive	200, 400, and 600 μg/ml	Single Bond 2	MTBS
Gotti VB	2015	Brazil	J Adhes Dent	Quercetin	Adhesive	5 wt%	Single Bond 2; Clearfil SE Bond (Kurary Noritake Dental; Tokyo, Japan); Easy Bond (3M ESPE, St. Paul, MN, United States)	MTBS
Du X	2012	China	J Dent	EGCG	Adhesive	100, 200, and 300 μg/ml	Single Bond 2	MTBS
Peng W	2020	China	Materials Science and Engineering C	Resveratrol	Primer	1, 10, and 20 μg/ml	Single Bond Universal	MTBS
Zhang Z	2020	China	Dental Materials	EGCG	Primer	0.01%, 0.1%, 1%	Single Bond Universal	MTBS
Landmayer K	2020	Brazil	J Prosthet Dent	EGCG; Proanthocyanidin (PA)	Primer	EGCG at 400 μM; 10% PA	Single Bond 2	MTBS
Dávila-Sánchez A	2020	Chile	Dent Mater	Quercetin; Hesperidin; Rutin; Naringin; Proanthocyanidin	Primer	0.065	Single Bond Universal	MTBS
de Siqueira FSF	2020	Brazil	Clin Oral Investig	Proanthocyanidin	Primer	0.065	Prime and Bond Elect (Dentsply Sirona, Milford, DE, United States); Single Bond Universal; Tetric n-Bond Universal (Ivoclar Vivadent AG, Schaan, Liechtenstein)	MTBS
Yi L	2019	China	J Dent	Baicalein	Primer	0.01%, 0.05%, and 1% w/v	Single Bond Universal	MTBS
Albuquerque N	2019	Brazil	Oper Dent	EGCG	Primer	0.1% EGCG; or 1% PLGA/EGCG	Single Bond 2	MTBS
Costa CAG	2019	Brazil	J Adhes Dent	EGCG	Primer	0.1% EGCG; or 2% CHX	Clearfil SE Bond	MTBS
Fialho MPN	2019	Brazil	J Mech Behav Biomed Mater	EGCG	Primer	0.02%; 0.2%; 0.5%	Single Bond 2	MTBS
Li J	2018	China	Oper Dent	Baicalein	Primer	0.1, 0.5, 2.5, and 5.0 μg/ml	Single Bond 2	MTBS
Porto ICCM	2018	Brazil	Eur J Oral Sci	Quercetin; Resveratrol	Primer	100, 250, 500, or 1,000 μg ml, a mixture of quercetin and resveratrol (3:1, 1:1, 1:3; vol:vol	Single Bond Universal	MTBS
Bacelar-Sá R	2017	Brazil	Braz Dent J	Proanthocyanidin	Primer	0.065	Single Bond Universal; Prime and Bond Elect; All-Bond 3 (Bisco Inc., Schaumburg, IL, United States); G-Aenial (GC Corp., Tokyo, Japan)	MTBS
Li K	2017	China	RSC Adv	Quercetin	Primer	0.1, 0.5, and 1 wt%	Single Bond 2	MTBS
Zheng P	2017	China	Sci Rep	Proanthocyanidin	Primer	0.05	Single Bond 2	MTBS
Zhou J	2016	China	Dent Mater	Grape seed extract	Primer	5 mass%	Single Bond 2	MTBS
Hass V	2016	Brazil	Dent Mater	Proanthocyanidin	Primer	6.5 wt%	Single Bond Plus; Tetric N-Bond	MTBS
Yang H	2016	United States	J Dent	EGCG	Primer	0.02% and 0.1%	Single Bond 2	MTBS
Zheng P	2015	China	Oper Dent	Grape seed extract	Primer	0.0005	OptiBond FL (Kerr, Scafati, Italy); Clearfil SE Bond	MTBS
Islam MS	2014	Japan	Dent Mater	Proanthocyanidin; Hesperidin	Primer	0.5%, 1%, 2%, 5% of hesperidin (HPN) or 0.5% of proanthocyanidins (PA)	Clearfil SE Bond	MTBS
Liu RR	2014	China	Int J Oral Sci	Proanthocyanidin	Primer	10% or 15%	Single Bond 2	MTBS
Santiago SL	2013	Brazil	J Adhes Dent	EGCG	Primer	0.02%, 0.1%, or 0.5% w/v	Single Bond 2	MTBS
Broyles AC	2013	United States	J Prosthodont	Grape seed extract	Primer	0.065	RelyX Unicem (3M ESPE, St. Paul, MN, United States); G-Cem self-adhesive cements (GC America, Alsip, IL)	MTBS
Liu RR	2012	China	Zhonghua Kou Qiang Yi Xue Za Zhi	Proanthocyanidin	Primer	0.15	Single Bond 2	MTBS
Macedo GV	2009	United States	J Dent Res	Grape seed extract	Primer	0.065	Single Bond 2; One Step Plus (Bisco, Schaumburg, IL, United States)	MTBS
Al-Ammar A	2009	United States	J Biomed Mater Res B Appl Biomater	Grape seed extract; Genipin	Primer	6.5% GSE; 0.5% GE	One Step Plus; Single Bond Plus	MTBS

Abbreviation: EGCG, epigallocatechin-3-gallate; EGCG-3Me, epigallocatechin-3-O-(3-O-methyl)-gallate; GSE, grape seed extract; GE, genipin.

### Quality Assessment

Two reviewers (SZ and HY) independently assessed the risk of bias of the included studies with the assessment instrument used in a previous systematic review of *in vitro* studies (Sarkis-Onofre et al., 2014). Quality assessment parameters included randomized teeth, teeth free of caries or restoration, operation following the manufacturer’s instructions, given sample size, and the bonding procedures were performed by a single operator with or without blinding. The article would be given a “Yes” on the parameter if it was reported and performed appropriately in the article; and a “No” if it was not mentioned or not performed properly. Articles were classified into three levels of risk of bias according to the number of parameters that scored “Yes”: high (≤2 parameters), medium (3-4 parameters), and low (5-6 parameters) (Table 2).

**TABLE 2:** Risk of bias of the studies considering aspects reported in the Materials and Methods section.

Study	Year	Random	Caries	Adhesive	Sample	Operator	Blind	Risk
Peng W	2020	Y	Y	Y	Y	Y	N	Low
Zhang Z	2020	Y	N	Y	Y	Y	N	Medium
Landmayer K	2020	N	Y	Y	Y	N	N	Medium
Dávila-Sánchez A	2020	Y	Y	Y	Y	Y	N	Low
de Siqueira FSF	2020	Y	Y	Y	Y	Y	N	Low
Albuquerque N	2019	Y	Y	Y	Y	N	N	Medium
Yi L	2019	Y	Y	Y	Y	Y	N	Low
Albuquerque N	2019	N	Y	Y	Y	N	N	Medium
Costa CAG	2019	Y	Y	Y	Y	N	N	Medium
Fialho MPN	2019	N	Y	Y	Y	Y	N	Medium
Li J	2018	Y	Y	Y	Y	N	N	Medium
Porto ICCM	2018	Y	Y	Y	Y	N	N	Medium
Yang H	2017	Y	Y	Y	Y	N	N	Medium
Yu HH	2017	Y	Y	Y	Y	N	N	Medium
Bacelar-Sá R	2017	N	Y	Y	Y	N	N	Medium
Li K	2017	Y	Y	Y	Y	N	N	Medium
Zheng P	2017	N	Y	Y	Y	N	N	Medium
Zhou J	2016	N	Y	Y	Y	N	N	Medium
Hass V	2016	N	Y	Y	Y	N	N	Medium
Yang H	2016	N	Y	Y	Y	Y	N	Medium
Gotti VB	2015	Y	N	Y	Y	N	N	Medium
Zheng P	2015	Y	Y	Y	Y	N	N	Medium
Islam MS	2014	N	Y	Y	Y	N	N	Medium
Liu RR	2014	Y	Y	Y	Y	N	N	Medium
Santiago SL	2013	N	Y	Y	Y	N	N	Medium
Broyles AC	2013	Y	Y	Y	Y	N	N	Medium
Du X	2012	Y	N	Y	Y	N	N	Medium
Liu RR	2012	Y	Y	Y	Y	N	N	Medium
Macedo GV	2009	Y	Y	Y	Y	N	N	Medium
Al-Ammar A	2009	Y	Y	Y	Y	N	N	Medium

### Statistical Analysis

Meta-analysis was conducted using Review Manager 5.3. Each possible comparison of the bond strength of dental adhesives with or without plant extracts participation was undertaken. In order to minimize the heterogeneity, only *in vitro* studies comparing the same plant extracts with the same concentration was included in the global analysis. The mean difference with 95% confidence interval (CI) was calculated and *p* ≤ 0.05 was considered significant. Statistical heterogeneity was assessed using the modified chi-square test (Cochran’s Q), which indicates heterogeneity when p>0.1, and I^2^ test, which indicates heterogeneity when its values is greater than 50%. Random-effect model was used in the analysis. The publication bias was to be assessed if more than ten studies were included in a meta-analysis. Sensitivity analysis was also performed by sequentially excluding each study if there were sufficient studies (≥10).

## Results

### Search Strategy and Characteristics

**FIGURE 1:**
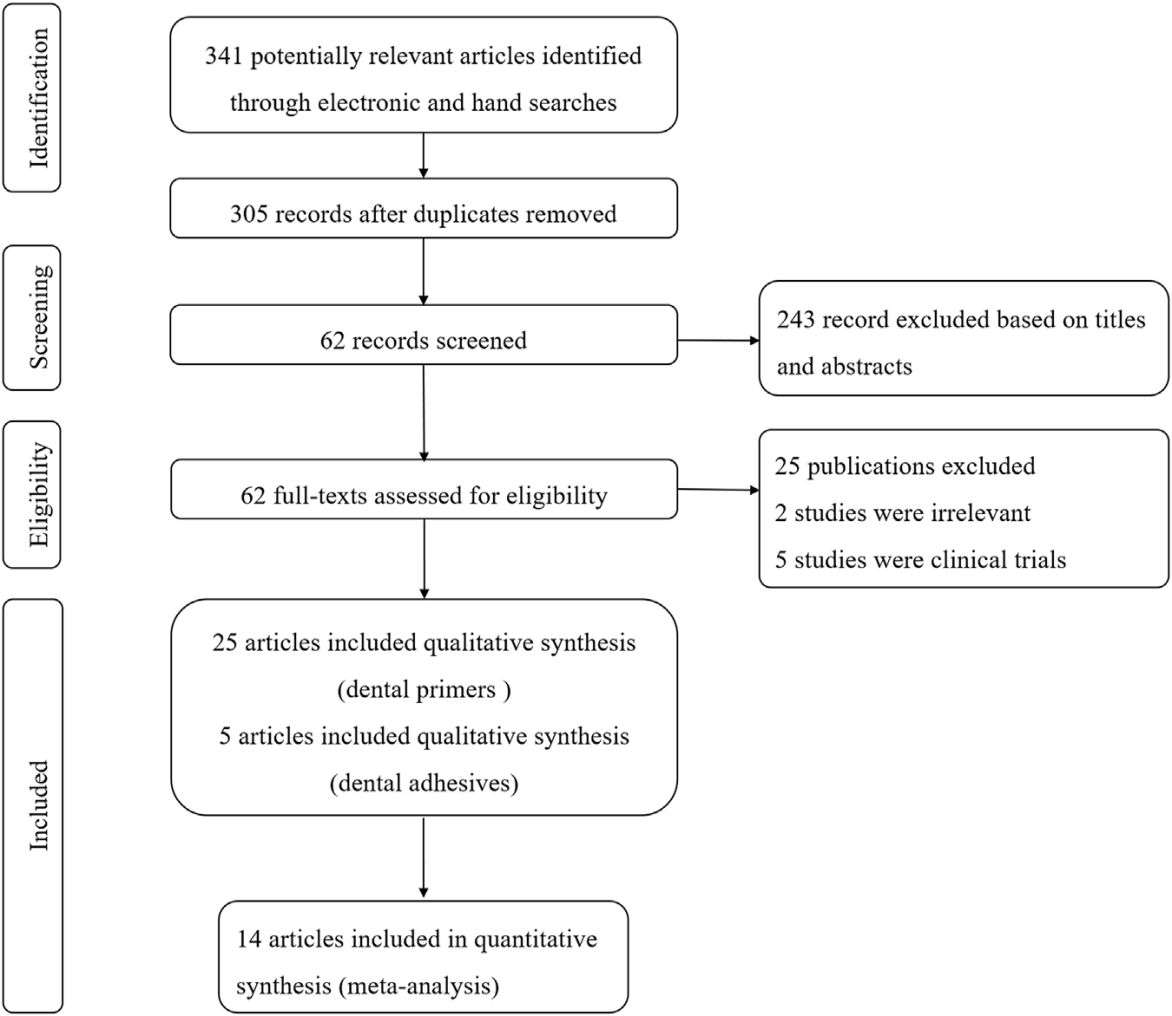
Flow chart of study selection according to PRISMA statement.

The initial search yielded 341 articles, out of which, 36 articles were eliminated after screening of titles and removal of duplicates. After abstract screening, 243 articles were excluded. A resultant sample of 62 articles was carried forward to the next stage, in which full-text copies were scrutinized. Finally, a total of 30 studies were systematically reviewed, in which 5 studies added plant extracts into adhesives and 25 studies used plant extract solution as primers (Figure 1). Twenty-nine articles were in English and 1 were in Chinese. There are nine types of plant extracts and 15 types of adhesives involved (Table 1).

### Risk of Bias

**FIGURE 2:**
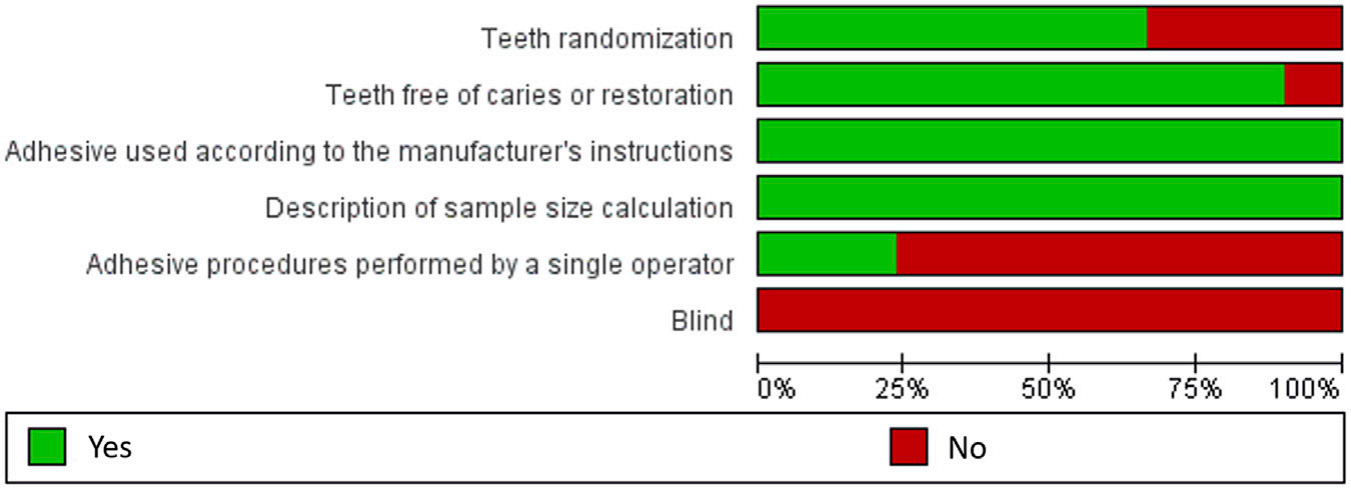
Risk of bias graph judgements about each risk of bias item presented as percentages across all included studies.

Most of the 30 studies (86.7%) exhibited a medium risk of bias, except for four (13.3%) with a low risk of bias. All of the studies used the adhesive according to the manufacturer’s instructions and described sample size calculation, but none of the studies reported blinding. A total of 20 studies (66.7%) reported random assignment of teeth, and 27 studies (90%) used teeth free of caries. Only seven studies (23.3%) reported adhesive procedure performed by a single operator. The results are described in Figure 2 and Table 2.

### Meta-Analysis

In the studies included in the meta-analysis, we only choose the data of interest. Only commercial adhesives were included, and the studies used experimental adhesives were excluded (Epasinghe et al., 2012). The effect of plant extracts on bonding strength may be related to different bonding modes such as self-etch or etch-and-rinse (Macedo et al., 2009; Bacelar-Sa et al., 2017). Hence, the disparity of the bond strength of different plant extracts in self-etch or etch-and-rinse adhesives was compared. Because aging methods were highly heterogeneous (i.e., water storage, saliva storage and PH cycling), it was not considered in the meta-analysis (Deng et al., 2014).

**FIGURE 3:**
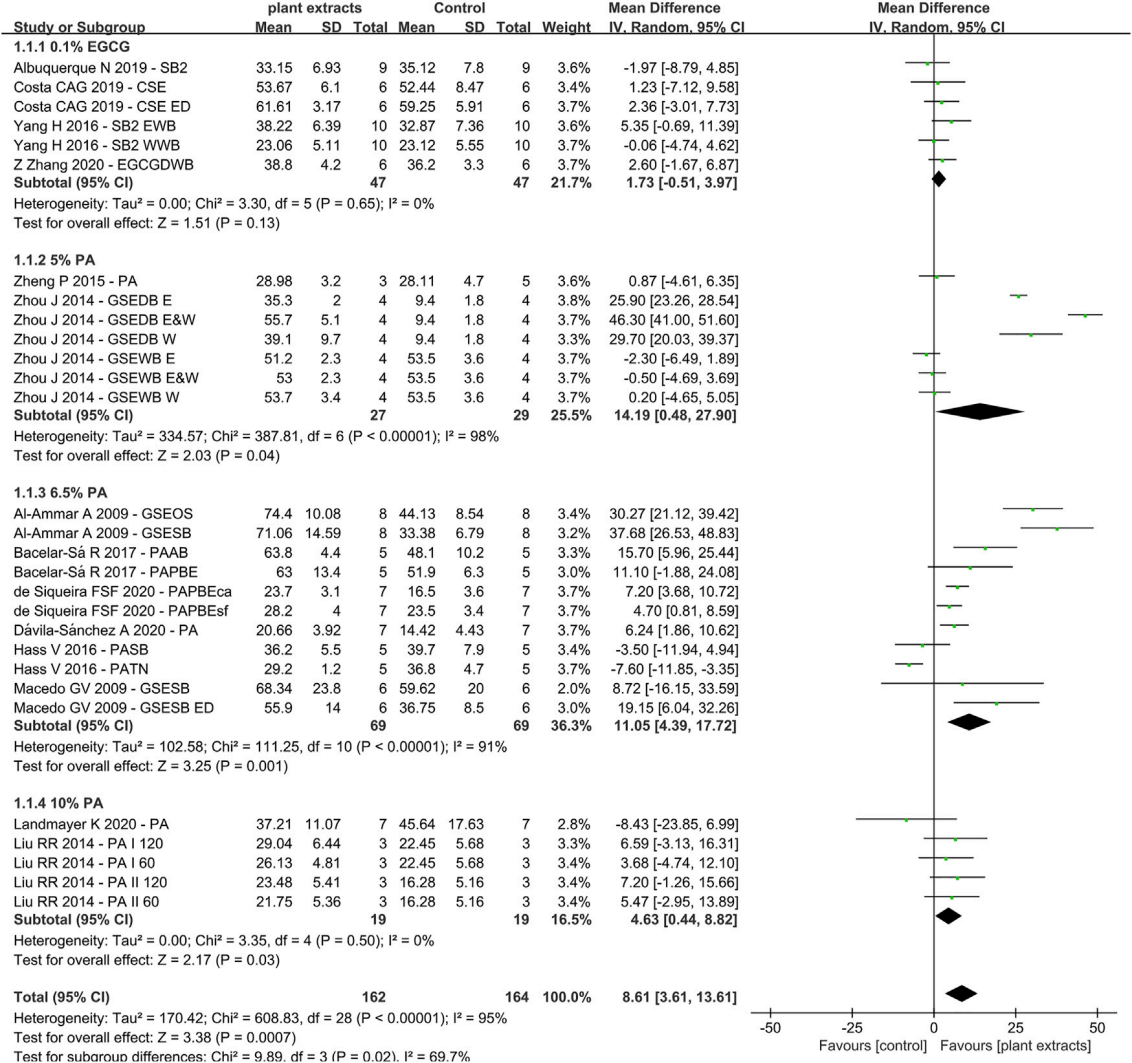
Forest Plot—plant extract primers: etch-and-rinse immediate bond strength.

**FIGURE 4:**
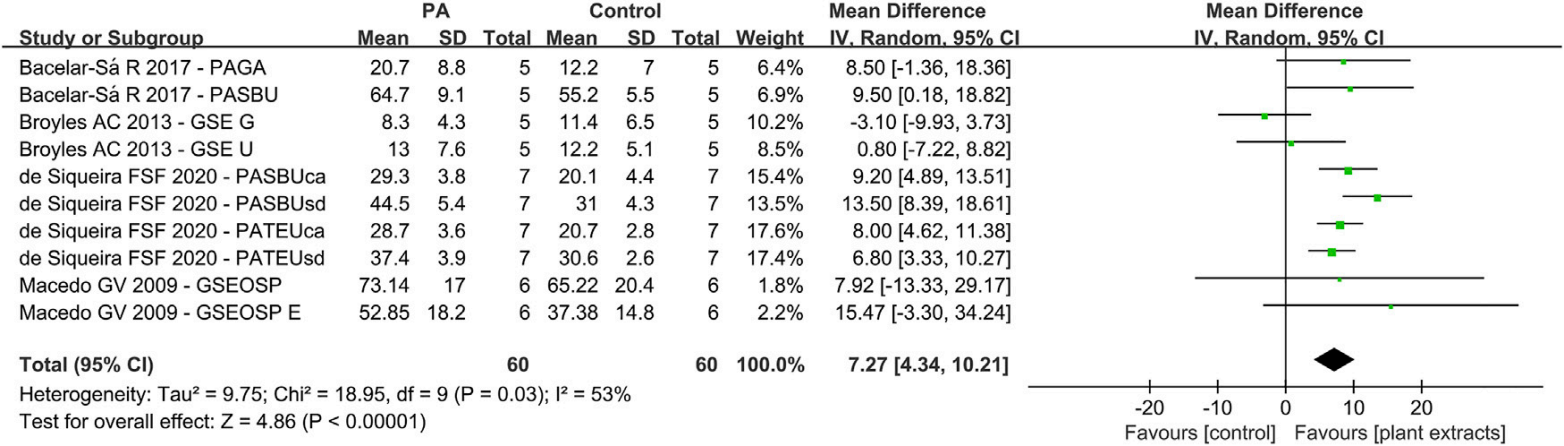
Forest Plot—plant extract primers: self-etch immediate bond strength.

**FIGURE 5:**
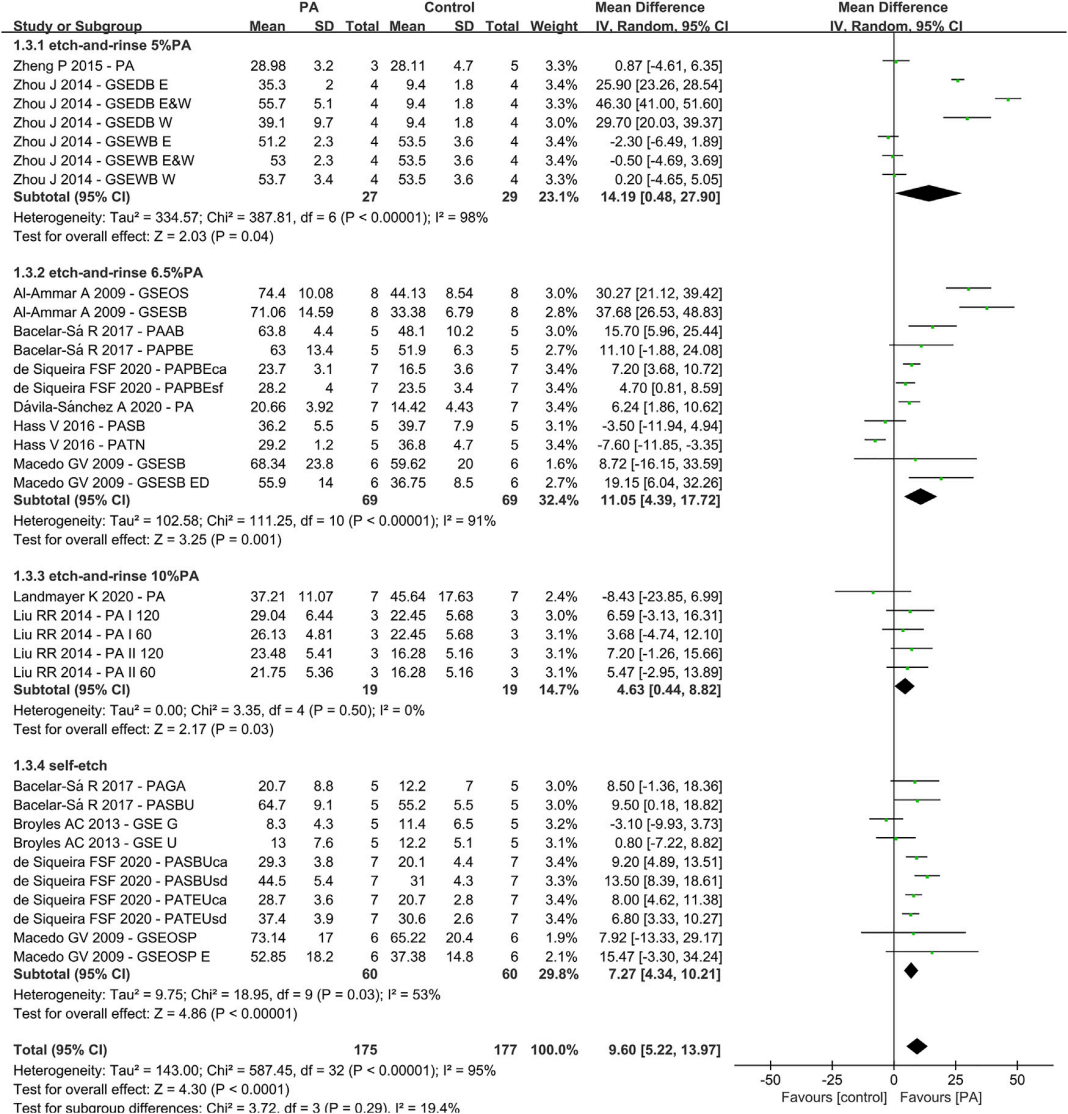
Forest Plot—proanthocyanidin (PA) primers

Due to the fact that different concentrations of plant extracts were used, only those with the same concentration were taken into meta-analysis. Of the 30 studies, data from 14 papers in which plant extract solution serve as primers underwent meta-analysis. The results of the meta-analysis are shown in Figures 3–5.

### Etch-and-Rinse Bond Strength (Plant Extract Primers)

The first analysis (etch-and rinse adhesive with or without plant extract primers) was performed, and the different concentration of plant extracts were the subgroups. A total of 29 datasets were selected, while 14 studies were included (Figure 3), with the following results: Q-test *p* < 0.00001, I^2^ = 95% and overall effect *p* = 0.0007. Test for subgroup differences: Q-test *p* = 0.02 and I^2^ = 69.7%, which showed that the data of subgroups were consistent.

The data of subgroup using 0.1% EGCG as primer showed no statistically significant differences compared with control group (Z-test: *p* > 0.05). However, the result of proanthocyanidin (PA) showed that the experimental groups had significant higher bond strengths than the control groups, with overall effect *p* < 0.05. For primers with 5% PA and 6.5% PA, the result in the Q-test was both *p* < 0.01 and I^2^ = 98%, I^2^ = 91%, separately. However, the result of 10% PA in the Q-test was *p* > 0.05 and I^2^ = 0%. The results of the meta-analysis are shown in Figure 3.

### Self-Etch Bond Strength (Plant Extract Primers)

For the second analysis (self-etch adhesive with or without plant extract primers), 10 data sets were selected, with four studies included (Figure 4). The results were as followings: Q-test *p* < 0.05 and I^2^ = 53%. The global analysis showed statistically significant difference (*p* < 0.05).

### Primers With Vs Without Proanthocyanidin

For the third analysis (primers with or without PA), 11 studies and 33 datasets were included (Figure 5). The difference between control and experimental groups were statistically significant (Q-test: *p* < 0.01, I^2^ = 95% and Z-test: *p* < 0.05). The differences in the test for subgroups (primers with different concentration of PA) showed the following values: chi-squared = 3.72, df = 3 (*p* = 0.29) and I^2^ = 19.4%. The meta-analysis results are shown in Figure 5.

## Discussion

This systematic review is the first to verify the effects of plant extracts on dentin bonding strength from *in vitro* studies. Thorough database research was conducted, and data were extracted and integrated in tables. Each study was designed and performed on the basis of their own parameters (plant extract types, action modes, concentration, dental adhesives and indicators), as listed in Table 1. Nine different plant extracts were added into 15 types of adhesives or served as primers. Out of the 30 studies, the data from 14 were selected for further evaluation.

As shown in Table 1, there were different commercial adhesives used. We had undertaken several measures to avoid the discrepancy. Firstly, the details of the adhesives were listed, such as commercial name, manufacturer, and place of production. Secondly, the articles that used experimental adhesives were excluded in the present study. Thirdly, 19 of 30 included studies chose the same one commercial adhesive, Single Bond 2(3M ESPE, St. Paul, MN, United States). Furthermore, all included studies set the control group which did not add plant extracts into adhesives or serve as primers. All these strategies were helpful to eliminate the disturbance of different adhesives on research results to the utmost extent. Furthermore, the included studies all reported the manufacturers and details of plant extracts, such as resveratrol powder (Sigma–Aldrich, St. Louis, MO, United States), and the pureness of this product was listed as ≥99% (HPLC).

Since plant extract was introduced, its effectiveness in crosslinking and biocompatibility has drawn a lot of attention. Different plant extracts were investigated, listing as follows: proanthocyanidin (PA), epigallocatechin-3-gallate (EGCG), quercetin (QUE), resveratrol (RSV), baicalein (BAI), hesperidin (HES), rutin (RUT) and naringin (NAR). Firstly, despite the chemical structure differences, they all belong to plant polyphenol, which possesses antioxidant and anti-inflammatory properties. These effects are mainly derived from phenolic hydroxyl groups in polyphenols (Leopoldini et al., 2011). The highly-hydroxylated structures make them capable of forming insoluble complexes with carbohydrates and proteins (Bravo, 1998; Teixeira et al., 2002). The major force that stabilizes the plant-extract-protein complexes is hydrogen bonding between phenolic hydroxyl and peptide carbonyl (Hagerman and Butler, 1980a; Hagerman and Butler, 1980b), which is strengthened by alkyl substitution on the amide nitrogen adjacent to the carbonyl (Cannon, 1955). Therefore, the mechanical properties of collagen complex would be increased (Yang et al., 2016). Secondly, plant extracts, such as baicalein and resveratrol, can inhibit the activity of peptidases directly or indirectly by changing the catalytic domain (Mazzoni et al., 2018) or crosslinking with noncollagenous proteins regulating peptidases (Breschi et al., 2010; Cova et al., 2011). Thirdly, many plant extracts, like baicalein, are commonly used in herbal medicines to treat bacterial and viral infections. They show remarkable antimicrobial effects on different bacteria including *Escherichia coli*, *P. cuspidatum* and *S.mutans* (Song et al., 2006; Duan et al., 2007; Zeng et al., 2008; Chinnam et al., 2010; Jang et al., 2014). The mechanisms are not clear yet, but it might be attributed to the inhibition of the cellular growth (Paulo et al., 2010).

One of the most studied plant extracts is proanthocyanidin (PA), also known as grape seed extracts (GSE) (Al-Ammar et al., 2009; Macedo et al., 2009; Liu et al., 2012; Broyles et al., 2013; Liu et al., 2014; Islam et al., 2014; Zheng et al., 2015; Zhou et al., 2016; Hass et al., 2016b; Bacelar-Sa et al., 2017; Zheng and Chen, 2017; de Siqueira et al., 2020; Ds et al., 2020; Landmayer et al., 2020). It is a condensed tannins extracted from *Vitis vinifera* grapes, which has been reported to contain 79.6% polyphenols(Aguiar et al., 2014). PA is composed of flavon-3-ol subunits, catechin, epicatechin and epicatechin-3-O-gallate and linked through C4-C8 (Cavaliere et al., 2010). These components are responsible for their properties such as free-radical scavenging capacity, high affinity for protein, antioxidant potential and capacity to enhance the mechanical properties of collagen (Castellan et al., 2010; Leme-Kraus et al., 2017). Epasinghe et al. (2012) reported that incorporation of less than 3% proanthocyanidin into dental adhesive can reduce nanoleakage without comprising 24 h adhesive-dentin bond strength. The meta-analysis of the PA primer effects on bonding showed a significant positive effect compared with the control group, irrespective of the concentrations or the type of adhesive used (Al-Ammar et al., 2010; Macedo et al., 2009; Liu et al., 2014; Wiegand et al., 2015; Zhou et al., 2016; Hass et al., 2016a; Bacelar-Sa et al., 2017; Ds et al., 2020; Landmayer et al., 2020; Siqueira et al., 2020). However, the results of 5 and 6.5% PA primer revealed a heterogeneity of 98% and 91% (Figure 3). The reason might be attributed to different bonding techniques such as dry bonding and wet bonding (Zhou et al., 2016) For 10% PA primer, the bonding strength shows statistically significant elevation with no heterogeneity (Liu et al., 2014; Landmayer et al., 2020). Although the heterogeneity varies from group to group, the subgroup analysis revealed no significant differences, which also prove the effectiveness of PA primer.

Another important plant extract being intensely investigated is epigallocatechin-3-gallate (EGCG) (Du et al., 2012; Santiago et al., 2013; Yang et al., 2016; Yu et al., 2017; Albuquerque et al., 2019; Costa et al., 2019; Fialho et al., 2019; Landmayer et al., 2020; Zhang et al., 2020). It is one of the flavanols in tea, also known as catechins (Tachibana, 2011). As a representative component of green tea, it cannot be found in any plants except *C. sinensis (L.)* Kuntze (Tachibana, 2011). EGCG consists of a meta-5,7-dihydroxyl-substituted A ring and trihydroxy phenol structures on both the B and D rings (Peter et al., 2017). The polyphenolic structure makes EGCG good donors for hydrogen bonding (Yang et al., 2009). Thus, it has shown the ability to bring various health benefits, like anti-metastasis, anti-inflammatory and antioxidant effects (Mukhtar and Ahmad, 2000; Mereles and Hunstein, 2011; Suzuki and Isemura, 2013). The similarity of chemical structure with other flavanols like PA makes it capable of enhancing the mechanical strength of collagen. The addition of EGCG directly into adhesives has been proven to preserve the bond strength after different ageing methods (Du et al., 2012; Yu et al., 2017; Albuquerque et al., 2019). The result of EGCG primer showed no negative influence on immediate bond strength (Zhang et al., 2020). The lack of data and various ageing methods make it impossible to do meta-analysis on aged bond strength. However, plenty of articles showed EGCG primer can improve the bond stability (Landmayer et al., 2020; Zhang et al., 2020). Furthermore, Yu et al. (2017) created a derivative of EGCG, called EGCG-3Me, which can enhance the bond stability, inhibited *S.mutans* adhesion and hinder its growth.

There are other plant extracts included in this systematic review: quercetin (QUE) (Gotti et al., 2015; Yang et al., 2017; Ds et al., 2020), resveratrol (RSV) (Porto et al., 2018; Peng et al., 2020), baicalein (BAI) (J. Li et al., 2018; Yi et al., 2019), genipin (GEN) (Al-Ammar et al., 2009), hesperidin (HES) (Islam et al., 2014; Ds et al., 2020), rutin (RUT) (Ds et al., 2020), and naringin (NAR) (Ds et al., 2020). The molecular formula, mass and number of hydroxyphenyl radicals are listed in Table 3. The data are inadequate to perform meta-analysis.

**TABLE 3:** Physical and chemical properties of the plant extracts and their possible effects on immediate bonding strength.

Plant extracts	Molecular formula	Mol. Weight (g/mol)	Number of hydroxyphenyl radicals	Effects on immediate bonding strength
Proanthocyanidin	C_30_H_26_O_13_	594.5	7	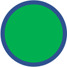
Epigallocatechin gallate	C_22_H_18_O_11_	458.4	8	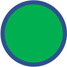
Quercetin	C_15_H_10_O_7_	302.2	5	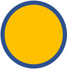
Resveratol	C_14_H_12_O_3_	228.2	3	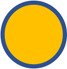
Baicalein	C_15_H_10_O_5_	270.2	3	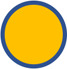
Genipin	C_11_H_14_O_5_	226.2	2	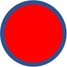
Hesperidin	C_28_H_34_O_15_	610.6	2	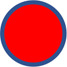
Rutin	C_27_H_30_O_16_	610.5	4	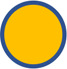
Naringin	C_27_H_32_O_14_	580.5	2	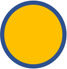

Green = evident; Yellow = unclear; Red = not recommended.

As natural crosslinkers, there are many factors influencing the crosslinking process. For instance, 1) the molecule size; 2) the number of molecules available in the solution; 3) the solubility index of the molecule and its influence on the miscibility of the vehicle for its application in dentin; 4) the number and type of reactive sites of the molecule; 5) the characteristics of the dentin (Ds et al., 2020).

It is a paradox that the bigger molecules usually have more reactive sites that can enhance the crosslinking effect, but their ability to dissolve and diffuse would be lower than smaller ones. Moreover, the type of molecules in grape seed extracts are complex, with monomers, oligomers and polymers existing at the same time (Bravo, 1998). The size of the oligomers and polymers were larger, which makes it more difficult to diffuse into dentin tubules. According to the results of included studies, we concluded the possible effects of different plant extracts on immediate bonding strength and classified them into different colors: green means the effects on improving bonding strength were evident; yellow means more studies in need; red means probable adverse effects (Table 3).

The plant extracts are normally recognized as plant polyphenols, which encompass a wide variety of molecules that contain at least one aromatic ring with one or more hydroxyl groups (Ferrazzano et al., 2011). Although they were extracted from different plants, the similarity in their chemical structure makes it possible for them to all possess properties like antioxidation and anti-bacterium. To begin with, the highly-hydroxylated structures make them capable of forming complexes with proteins, especially proline-rich proteins in dental collagen (Bravo, 1998). This fortified crosslinking interaction helps enhance the mechanical strength of dental bonding (Yang et al., 2017; Yi et al., 2019; Peng et al., 2020). Furthermore, the polyphenolic compounds could coordinate with metal ions and compete with peptidases such as MMPs for the catalytic domain in collagen (Mazzoni et al., 2018). As a result, the enzymatic hydrolysis of hybrid layer collagen would be impeded and the adhesive-dentin interface stability would be maintained (Epasinghe et al., 2012; Yang et al., 2016; Yang et al., 2017). Besides, the plant polyphenols were considered metabolites involved in the chemical defense of plants and possess the ability to inhibit bacteria (Ferrazzano et al., 2011). There is plenty of evidence supporting the inhibition of cariogenic bacteria by phenolic compounds. The mechanisms of polyphenols against bacteria like *S.mutans* may include affecting cell membrane permeability, inhibiting protein synthesis, blocking ATP synthesis and inhibiting bacterial metabolism (Chinnam et al., 2010; Xie et al., 2015). Lastly, as natural crosslinkers, the plant polyphenols are non-toxic compared to synthetic compounds like chlorhexidine and glutaraldehyde. They can protect cells by inhibiting oxidative stress-induced DNA damage, lipid peroxidation and protein oxidation (Kang et al., 2012). To conclude, all these *in vitro* studies demonstrated that the plant extracts, consisting of polyphenols, can enhance mechanical strength of dentin collagen, maintain dentin-adhesive stability, inhibit cariogenic bacteria and resist adhesive-induced cytotoxicity.

Although plant extracts have shown plenty of advantages, there are still a large variety of aspects to be explored, such as solvent, treatment time and concentrations. First, theoretically, the effect of plant extracts would increase with the concentration. However, the solubility of the compounds were not great (Bravo, 1998). Zhang et al. (2020) reported EGCG with dimethyl sulfoxide as a solvent can exert synergistic effect on dentin-adhesive interface stability. Second, the treatment time varies from one to another. Genipin is reported to have a slow rate of cross-linking induction that the mechanical strength increased only after 40 h treatment (Bedran-Russo et al., 2007). Third, the effect of different concentration on bonding is complex. It has been shown more than 3% PA added into adhesive directly can exert adverse effect on bonding (Epasinghe et al., 2012). More studies are needed to determine the suitable solvent, treatment time and concentrations of plant extracts.

As mentioned in this review, plant extracts are actually polyphenols, which possess phenolic hydroxyl groups and aromatic rings (Ferrazzano et al., 2011). Therefore, their solubility in solvents such as ethanol are high, due to their similar chemical structure like hydroxyl groups. Furthermore, the interactions between plant extract (eg. PA) and collagen can be disrupted by detergents of hydrogen bond-weakening solvents, suggesting that PA-collagen complex formation involves primarily hydrogen bonding between the protein amide carbonyl and the phenolic hydroxyl (Hagerman and Klucher, 1986). Ethanol, on the other hand, stimulate PA and collagen interactions (Bo et al., 2010). There is no evidence that the interaction is concentration-dependent.

The present study showed the changes in dentin bond strength after adding plant extracts into adhesives or serving as primers. Although strict selection was performed to minimize heterogeneity, the data of several subgroups remained high heterogeneous. There are three reasons for heterogeneity:1. Different adhesive brands; 2. Different bonding modes (etch-and-rinse or self-etch); 3. Different dentin material (normal or eroded dentin). Also, several authors failed to report important details, such as whether the same operator performed the bonding steps of all specimens. These factors may help explain the high heterogeneity in in vitro experiments.

## Conclusions

Plant extracts have positive effects on the immediate microtensile bond strength of the adhesive-dentin interface. Meta-analysis demonstrated that the use of proanthocyanidin (PA) primer, especially at the concentration of 10%, had statistically significant effect on the immediate dentin bonding strength. Considerable heterogeneity existed among the different adhesive brands, bonding modes and dentin materials used, which limited the meta-analysis approach. Further clinical research is needed to confirm the effect of plant extracts on bond strength *in vivo.*

## Data Availability

The original contributions presented in the study are included in the article/Supplementary Material, further inquiries can be directed to the corresponding authors.

## Appendix

**Table A1:** Search strategy used for PubMed (from inception to September 2021).

	Search terms
#1	Epigallocatechin gallate OR epigallocatechin-3-gallate OR epigallocatechin-3-O-gallate OR “EGCG cpd” OR epigallo-catechin gallate
#2	Quercetin[MeSH]
#3	Genipin
#4	Proanthocyanidins[MeSH] OR “condensed tannin*” OR “anthocyanidin polymer*” OR procyanidin*
#5	Naringenin
#6	Hesperidin[MeSH] OR “hesperetin 7 rhamnoglucoside” OR “hesperetin 7 rutinoside”
#7	“Crosslinking agent*” OR “cross link*”
#8	Plant extracts[MeSH]
#9	#1 OR #2 OR #3 OR #4 OR #5 OR #6 OR #7 OR #8
#10	Dental cements[MeSH] OR “dental adhesive*” OR “luting agent*” OR dentistry [MeSH]
#11	Bond* AND strength
#12	#9 AND #10 AND #11
